# Modified bentonite @ microwave for Mn(VII) removal with a simulation study

**DOI:** 10.1038/s41598-025-91906-z

**Published:** 2025-03-12

**Authors:** Hend S. Abu Salem, Safaa S. Hassan, Fatma A. Refay, Ziad K. Sulieman, Mohamed Youssef Mohamed, Ahmed R. Rabea, Nada S. Refaay, Salma Y. Abdel Moain, Mohamed El-Sayed Abdulrahman, Omar N. Radwan, Mahmoud A. Roshdy, Fathy M. Mohamed

**Affiliations:** 1 Geology Department, Faculty of Science, Cairo University, Giza, 12613 Egypt; 2 Chemistry Department, Faculty of Science, Cairo University, Giza, 12613 Egypt; 3 Chemistry and Microbiology Department, Faculty of Science, Cairo University, Giza, 12613 Egypt; 4 Petroleum Geoscience Department, Faculty of science, Cairo University, Giza, 12613 Egypt; 5 Biophysics Department, Faculty of Science, Cairo University, Giza, 12613 Egypt; 6 Biotechnology Department, Faculty of Science, Cairo University, Giza, 12613 Egypt; 7 Faculty of Earth Sciences, Beni-Suef University, P.O. 62521, Beni-Suef, Egypt

**Keywords:** Adsorption, Bentonite, Manganese, Microwave, Valorization, Water treatment, Environmental sciences, Chemistry, Materials science

## Abstract

A reasonably priced and easily available natural bentonite was used to remove Mn(VII) ion from polluted water. The purpose of this research is to investigate the structural and adsorption capacity changes of microwave-treated bentonite following Mn(VII) ion adsorption. The two forms of bentonite (natural and microwave-assisted) were characterized with respect to the chemical composition and structural morphology (XRD, EDX and FTIR) in addition to pore size distribution and surface area. The structure of the microwave-treated bentonite showed partial damage of the framework of silica, and new surface nucleation centers are developed during microwave treatment. Montmorillonite was deemed to be the prevailing phase. The total surface area and the average pore size distribution were changed from (277,624 m^2^/g and 4,9118 nm) to (327,085 m²/g and 4.1691 nm) after microwave treatment. BET surface area expanded, hence enhancing the adsorption ability of Mn(VII) ions approximately 18.0% more than that of the untreated bentonite B. FTIR and SEM morphology pointed out the Mn(VII) adsorption ions onto the microwave-treated bentonite.

## Introduction

Water deterioration by lethal substances is a major problem as it negatively affects the health of humans, plants, animals, and the environment. Natural materials are commonly used to remove heavy metals from water due to their cost-effectiveness, plausibility, and ease of use. Natural resources, industrial waste, and agricultural wastes all serve as economical sorbents for heavy metal removal. These materials’ excellent selectivity, high stability, and mechanical strength behavior make them appropriate for application in water treatment^1^.

The environment and numerous ecosystems are at jeopardy due to heavy metal deposition in surface water caused by electroplating, the manufacture of plastics, fertilizers, pigments, mining, and metallurgical operations Furthermore, wastewater produced by chemical manufacturers is regarded as one of the primary sources of environmental pollution^2^, especially if it is reused for irrigation without being treated. Several inorganic and organic contaminants found in wastewater pose a health risk when they get into the food chain^3^.

As a result, elevated metal ion concentrations have been related to several harmful health effects, including liver and kidney damage, birth defects, lung and digestive tract cancer, skin lesions, retardation leading to impairments, and a host of other issues^4^. Among the most prevalent contaminants detected in effluents from chemical facilities are hazardous metal ions, such manganese^5^. For instance, despite the fact that the permissible limit of Mn ions level in household water is 0.4 ppm according to minster of health decree no. 2007, children and adults who consume water with high manganese levels over a lengthy period of time may experience problems with attention and memory issues^6^. Several methods were used to remove heavy metals, e.g., clinoptilolite has the potential to remove metals such as Pb (II), Cd(II), Zn(II) and Cu due to its ion exchange ability^7^. Clay, babel, and other materials^8–10^ are also used. The large surface area (approximately 200 m^2^/g) and high porosity of this material can effectively remove heavy metals from wastewater. 128 mg Cr^6+^/g peat moss was proven as its Cr^6+^ adsorption capacity in the pH range 1.2–2.7. Chitosan can remove heavy metals due to its chelating ability. Used to treat Hg^2+^, Cu^2+^, Ni^2+^, Zn^2+^, Cr^6+^, Cd^2+^, Pb^2+7^, rice husks^11,12^, black bean pods^13^, tea husks, seed pods^14^, papaya tree^15^, maize leaves^15^, teak leaves powder^16^, Coriandrum sativum^17^, peanut shell pellets^18^, sorghum palm waste^19^, strawberry leaves^20^.

Anthracite coal and carbon nanotubes (MWCNT) created for wastewater treatment have been constructed by many studies^21–23^. Activated silica^24^, olive granules^25^, combinations of anthracite and chitosan^26^, aluminum and iron coagulants coated with activated silica^27,28^, and PFTE derivatives^29–31^ are examples of derivatives of acrylonitrile.

Sorption is one of the most leading ways to eliminate heavy metals and nonmetals using natural and chemical adsorbents^32^. For example, to remove chromium (VI) from water, artificially synthesized goethite and activated carbon, as well as a mixture of the two materials, have been used^33^. In addition, Ethiopian acid-activated bentonite has been used to remove sodium from high salinity water^34^. Atkovska et al.^35^ used natural bentonite to remove Fe(II) and Zn(II) ions from landfill leachate. Similarly, Akpomie and Dawodu^36^ used natural bentonite to remove Ni(II) and Mn(II) from the binary system, with an adsorption capacity of about 206 and 149 mg/g during a stirring time of 24 h. In addition, El-Aassar et al.^31^ used a mixture of bentonite-anthracite-Zetag to remove As(V) from wastewater. El-Aassar and Mohamed^21^ also used upgraded anthracite coal to adsorb Mn(VII) from aqueous solution in static and dynamic studies, with an adsorption capacity of approximately 555 mg/g. Further study on Mn(II) ion removal using iron-aluminum copolymers combined with activated silica produced from rice husks^24,27,28^ was made.Mn (VII) was also removed from aqueous solution using CNTs^37^. CNTs adsorb metal ions at extrinsic, internal, pathogenic, and interstitial sites due to their large surface area and high van der Waals binding energy^38^. Anion exchange has been used to treat permanganate-contaminated water^39^. In addition, activated orange peel powder has been used to remove manganese (VII) ions from aqueous solutions^40^. According to the above statements, the utilization of common materials as sorbents to remove unwanted chemicals such as heavy metals and unwanted colors is dominated by the removal efficiency and cost-effectiveness of the materials. The capacity for adsorption of natural adsorbents has been enhanced using a variety of methods. (e.g., microwave treatment and acidification).

Bentonite is one of the clay minerals because it is an inexpensive, naturally occurring, plentiful sorbent material, despite having somewhat poorer sorption properties than modified or manufactured compounds. Bentonite’s sorption activity is enhanced by pre-activation. In order to modify bentonite in a green way, this study uses microwave stimulation. The changed bentonite is subsequently utilized to remove Mn(VII) from wastewater. Since microwave stimulation avoids the need for extra chemical reagents and the construction of additional treatment stages, it is preferable to chemical modification.

The target of this work has been set. (1) transformation of bentonite (B) to developed bentonite (DB) by microwaves, (2) emphasis on the role of microwaves in improving bentonite removal efficiency after modification, (3) by the influence of pH, adsorbent dose, adsorbent concentration, speed and stirring time on adsorption properties of Mn(VII) onto DB, (4) confirmation of experimental results by a series of kinetic, thermodynamic and isothermal models. Finally, confirm the adsorption process using a simulation study.

## Materials & methods

This part showed the materials utilized to complete this study, as well as the methodologies applied.

### Bentonite

The adsorption studies used commercial bentonite acquired from AL-Shark company for treading as the precursor material.

### Chemicals

To create a 1000 ppm solution of Mn(VII), 0.7315 g of KMnO_4_ was dissolved in 250 ml of double-distilled water. Al-Naser Company provided HNO_3_ and NaOH for the initial pH adjustment. The microwave-treated bentonite’s point of zero charge was determined using laboratory-grade NaCl (MTB). For regeneration studies, 0.15 M HCl was utilized.

### Preparation of the microwave-treated bentonite (MTB)

The raw bentonite was crushed and sieved below 150 microns, without further preparation, 20 g were microwaved for 20 min at 800 W to produce microwave treated - bentonite (MTB). At 80 ° C, the sample was dried until its weight remained constant, then It had already it was packed and sealed. Using an M-10 magnetron, the authors established and developed a microwave generator. Using a normal switching circuit (without pulse modulation), the output power was 800 W and the radiation range was 2.45 GHz. The purpose of the horn antenna was to optimize radiation concentration and distribution.

### Materials characterization

Surface functional groups and micro-structural morphology of raw bentonite and MTB were investigated. Surface functional groups have been studied using a Fourier transform infrared spectrometer (FTIR) and were acquired using a test scan Shimadzu FTIR spectrometer (250–4000 cm^−1^) and the CsBr disc method. Scanning electron microscopy (SEM) investigation with a Quanta 250 FEG instrument was used to get the micro-structural morphology. Furthermore, utilizing a gas sorption instrument (Quantachrome, NOVA, version 11.04), SBET with N2 adsorption/desorption at 77 K has been carried out, and the amount of adsorbed N_2_ gas at P/P_o_ has been used to determine of the total pore volume (V_total_). In addition, the raw and MTB were exposed to a monochromatic X-ray beam with a varied incident angle range. The X-rays have been produced in a sealed tube with wavelength of 0.154 nm. The X-rays (Bruker LynxEye detector) have been detected by using a fast-counting detector based on silicon strip technology.

### Batch experiments

In the batch tests, 25 ml of 25 mg/L Mn(VII) were mixed with 50 mg MTB in clean Falcon™ 50 ml conical centrifuge tubes. The mixes were stirred (200 RPM) for 120 min, with an exception of the time of contact experiment.

The influencing elements were tested independently using Digital Shaker (DAIHAN-brand^®^ Precise Shaking Incubators, “ThermoStableTM IS-30) with initial concentrations (2–25) mg/L, different MTB dosages (5–50) mg, and different agitation speeds (100–200) RPM for (5–120) min. Using Whatman 32 filter paper, the remaining Mn(VII) solution was separated from the MTB. The concentrations of Mn(VII) in the filtrate solution was determined using an automated UV/Vis-NIR 3101 PC Shimadzu spectrophotometer at 525 nm. Table 1 summarizes the influencing factors. The removal (%) and adsorption capacity (mg/g) were calculated using Eqs. (1)& (2), where C_o_ (mg/l) and C_e_ (mg/L) are Mn(VII) in solution’s initial and equilibrium concentrations, respectively, and W (gram) and V (liter) are the mass of the MTB adsorbent and the volume of the Mn(VII) solution, respectively.

1 $$Removal= Co -Ce / Ce*100$$

2 $$qe = ( Co -Ce )^* V/M$$

**Table 1:** Factors that affect the adsorption of Mn(VII) upon MTB.

Factors	Conditions						
pH	3	5	7	8			
Dose (mg)	5	10	20	30	40	50	
Adsorbate concentration (mg/l)	2	5	10	15	20	25	
Contact time (min.)	5	15	30	45	60	90	120
Temperature (K)	288	303	313	318	333		
Agitation speed (RPM)	50	100	150	200			

### Characterization of adsorbent

#### Point of zero charge (pHpzc)

In a measuring flask, one-liter of a 0.01 M NaCl solution (0.585 g NaCl dissolved in one-liter distilled water) was prepared to establish the zero-charge point (pH_PZC_). The pH of nine falcon tubes containing 25 ml solution of 0.01 M NaCl have been adjusted for a number of starting pH (pH_i_) values (2–10) to 10 by adding 0.1 M solutions of either HCl or NaOH. After mixing these solutions with 50 mg of adsorbent, the suspensions have been shaken at 200 RPM for 2 h at room temperature, and after 24 h, the final pH (pH_f_) of each solution has been measured after 24 h^41^. The values of pH_i_ -pH_f_ vs. pH_i_ were shown on a graph (initial pH). The point when the pH of the solution changed to zero has been determined to be the intersection of pHi - pHf vs. pHi (where pHi = pHf), and this is the adsorbent’s pH_PZC_ value.

### Kinetic and isothermal and study of Mn (VII) sorption on MTB

Adsorption experiments were accomplished using various initial Mn(VII) concentrations in the isotherm study, and the optimum conditions of contact time, adsorbent dosage, and pH were determined. Langmuir, Freundlich, and Temkin isotherm models were used^42–44^. Each isotherm was used to assess the mechanism of Mn(VII) adsorption onto MTB. In addition, kinetic adsorption models such as (Lagergren et al., 1898 ^45^ & Ho & McKay 1999 ^46^) were used. The plot of the calculated theoretical values (q_e_) versus the equilibrium concentrations (C_e_) of Mn(VII) is used to calculate the model constants .

### Thermodynamic investigation of Mn(VII) adsorption onto MTB

Using Van’t Hoff’s equation, the enthalpy, entropy, and Gibbs free energy of Mn(VII) adsorption onto MTB were calculated.

### Reusability test

The ability of sorbent materials to be reused and reprocessed multiple times is an essential economic element since it affects production costs. The instantaneous attraction between the adsorbent surface and the metal ions was regained by regenerating the Mn-adsorbed MTB through desorption with 0.15 M HCl, washing with distilled water, and then applying the regenerated MTB for fresh Mn adsorption (VII). MTB reusability was determined following two successive cycles by comparing removal % and adsorption capacity at each cycle.

### Molecular dynamics (MD) simulation

The molecular dynamics (MD) simulation was performed using Material Studio 4.3 software (https://www.3ds.com/products/biovia/materials-studio) from Accelrys Inc. Bentonite (00–3, −112 and 111) planes were chosen for the simulation study. Adsorption Locator enables one to simulate a substrate loaded with permanganate ions. Adsorption Locator will be designed for the study of individual systems, allowing one to find low-energy adsorption.

## Results and discussion

### Identification of bentonite (B) and MTB

#### IR investigation

**Fig. 1:**
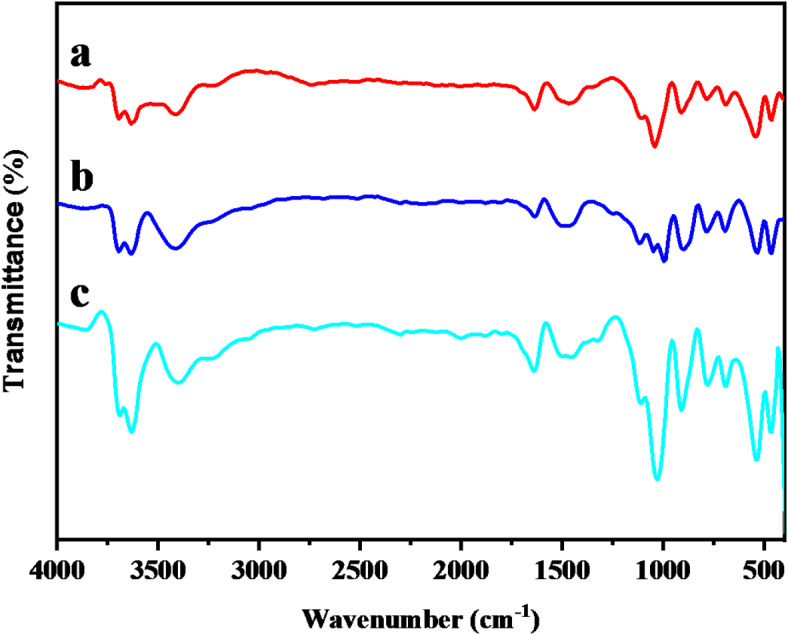
FTIR spectrum of (**a**) raw bentonite, (**b**) microwave-treated bentonite, (**c**) microwave-treated bentonite after Mn(VII) ion adsorption.

The spectra of FTIR related to B prior to and after treatment with microwave radiation and adsorption of Mn (VII) ions was shown in Fig. 1. According to numerous research, B is primarily made of metal oxides such as Al_2_O_3_, SiO_2_, MgO and CaO^47^ which can be characterized using FTIR. The FTIR spectra of B prior to microwave irradiation revealed an absorption peak at 3880 cm^–1^ (Fig. 1a), which corresponds to Al–OH–Al in the mineral^48^. The vibrations at 3633 cm^−1^ and 3695 cm^−1^ correlate to the -OH stretching (inner surface). The O–H stretching vibration of interlayer water molecules was observed as broadband at 3441 cm^−147,49^. The bands at 1126 –1049 cm^−1^ suggested the bending vibration of Si-O, whereas the stretching vibrations of Si-O were found around 794 –694 cm^−1^. The band at 918 cm^−1^ confirmed the presence of the Al-O bending vibration, whereas the absorptions at 547 –470 cm^−1^ correlate to the skeletal vibrations of Al-O-Si^50^.

After irradiating B with microwaves, we observed stronger peaks with minor shifts in certain bands, as seen in Fig. 1b. The Si–O bonds’ destruction led to increasing the number of free OH groups. The bending vibrational motion of Si-O was changed to a wider wavenumber at 1126 –995 cm^−1^. The observed signals at 786 –694 cm^−1^ were related to the stretching vibrations. At the lower wavenumber at 910 cm^−1^, the bending vibration of Al-O was found. Finally, the skeletal Al-O-Si building has become at 540 –470 cm^−1^. Several absorption shifts were observed after the adsorption of Mn (VII) ions (Fig. 1c); from (1651 cm^−1^ to 1643 cm^−1^), (3441 cm^−1^ to 3417 cm^−1^) and (2337 cm^−1^ to 2314 cm^1)^. The prior shifts were caused by the OH groups cooperating to adsorb the ion. The alterations of the Si-O group in absorption frequency from 1126 cm^−1^ to 1118 cm^−1^ and 1049 to 1033 cm^−1^ indicate their involvement in adsorption. The Al-O-Si linkage’s role in adsorption is also indicated by changing in absorption frequency from 547 cm^−1^ to 540 cm^−1^. The inner hydroxyl group in kaolinite is responsible for the O-H stretching band at 3621 cm^−1 51^.

#### XRD spectrum

**Fig. 2:**
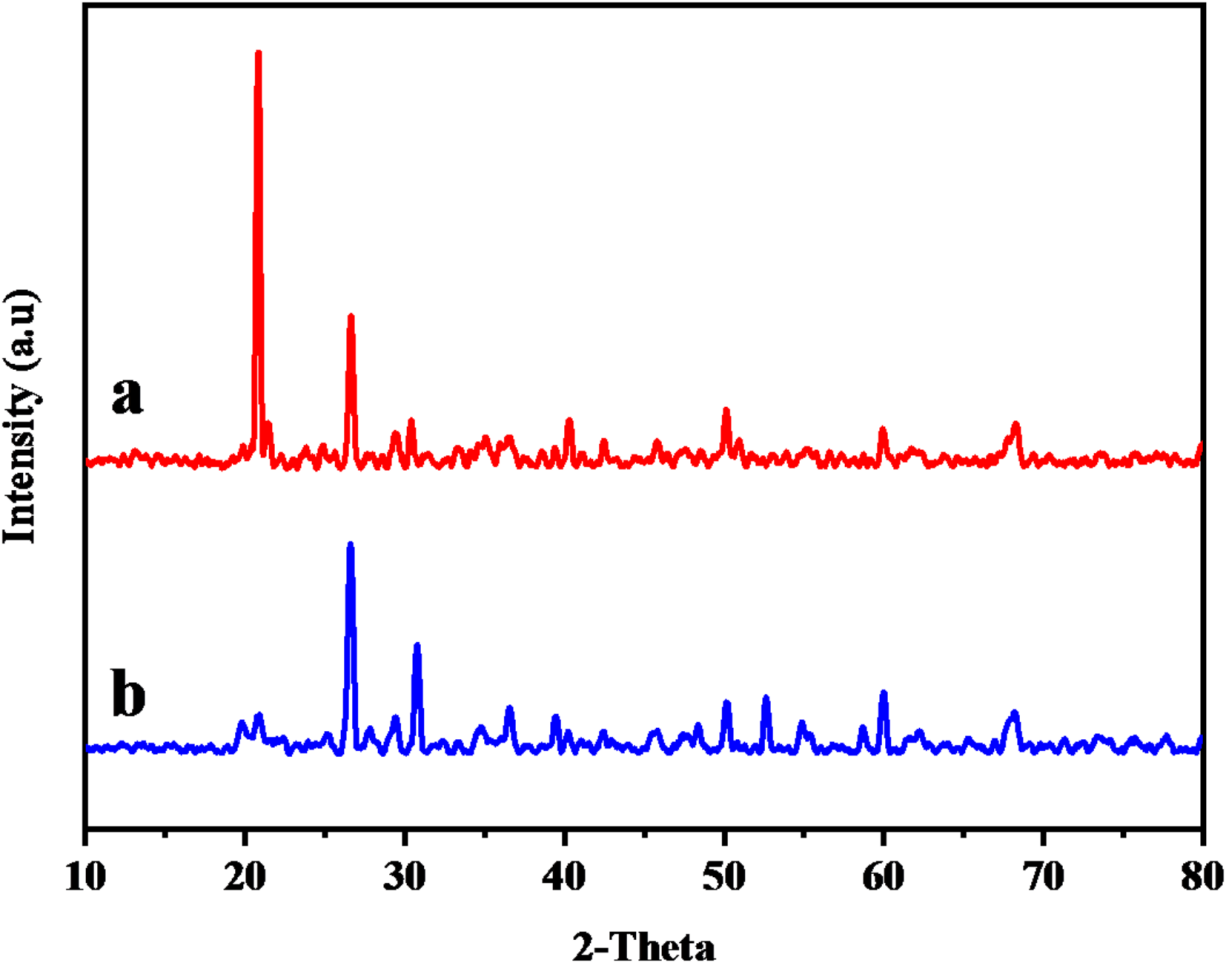
X-ray diffraction spectra: (**a**) Unmodified bentonite and (**b**) Bentonite under microwave radiation.

**Fig. 3:**
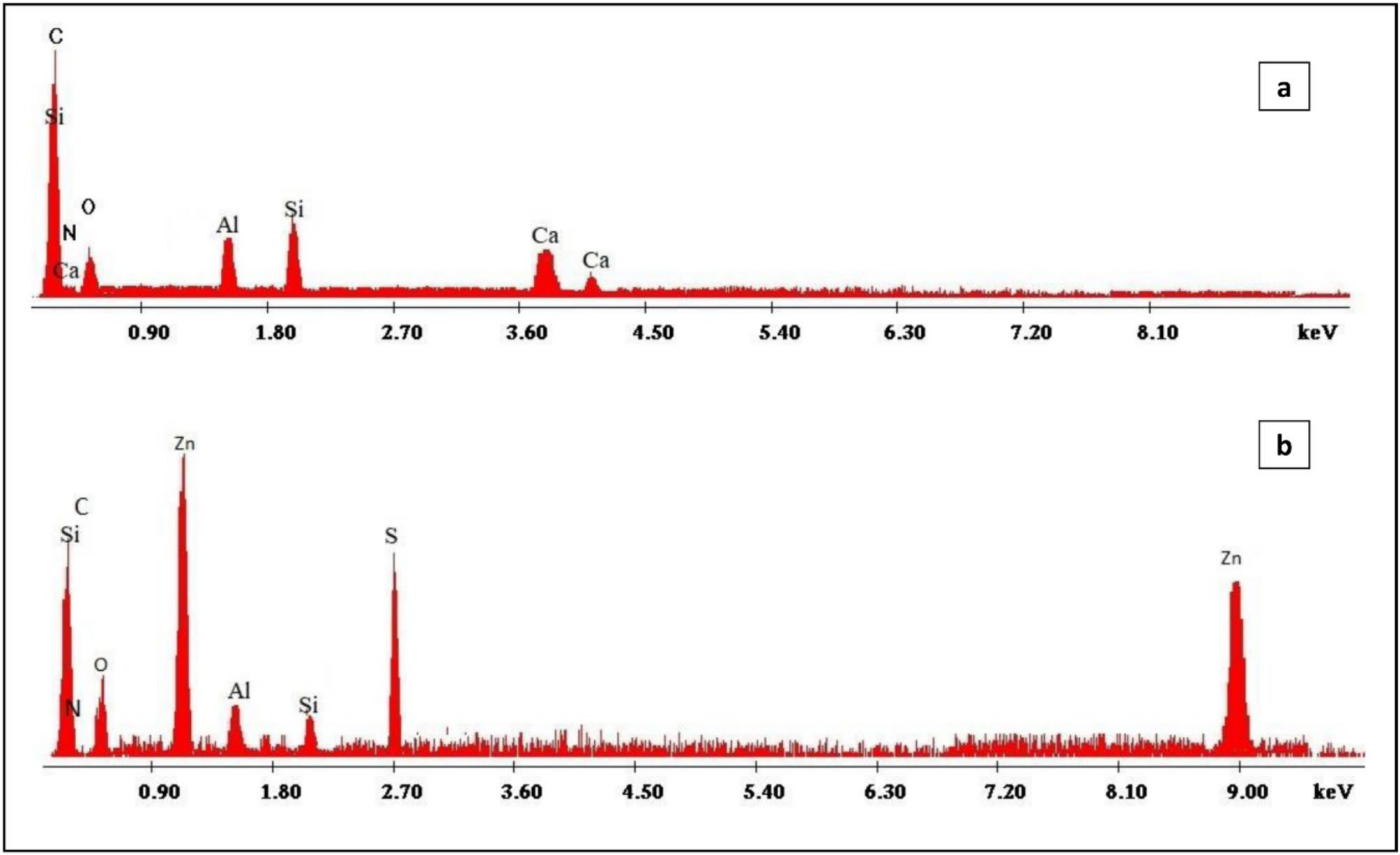
EDX spectra of (**a**) raw bentonite (B) and (**b**) microwave treated bentonite (MTB).

The XRD spectra of B and MTB (Fig. 2a **and b**) revealed a mineral composed of Montmorillonite with a triclinic crystal lattice. Al_2_ Ca_0.5_ O_12_ Si_4_ is the chemical formula a = 518,000, b = 898,000, and c = 1,500,000 are the dimensions. The existence of diffraction patterns related to angles at 5.887, 17.725, 19.757 and 23.080^o^ in the diffractograms confirmed the presence of montmorillonite in the materials^52^. Microwave activation enhanced and augmented the peak intensity due to the elimination of contaminants and dehydration, Si and K were the predominant elements in raw bentonite before and after treatment microwave irradiation, as determined by EDX spectral analysis (Fig. 3a **and b**). For bentonite prior to and after microwave treatment, the most prevalent compositions were (O K: 48.38 & Si: 40.18) and (O K: 42.09 & Si: 39.49), respectively.

#### Physical adsorption of N2

**Fig. 4:**
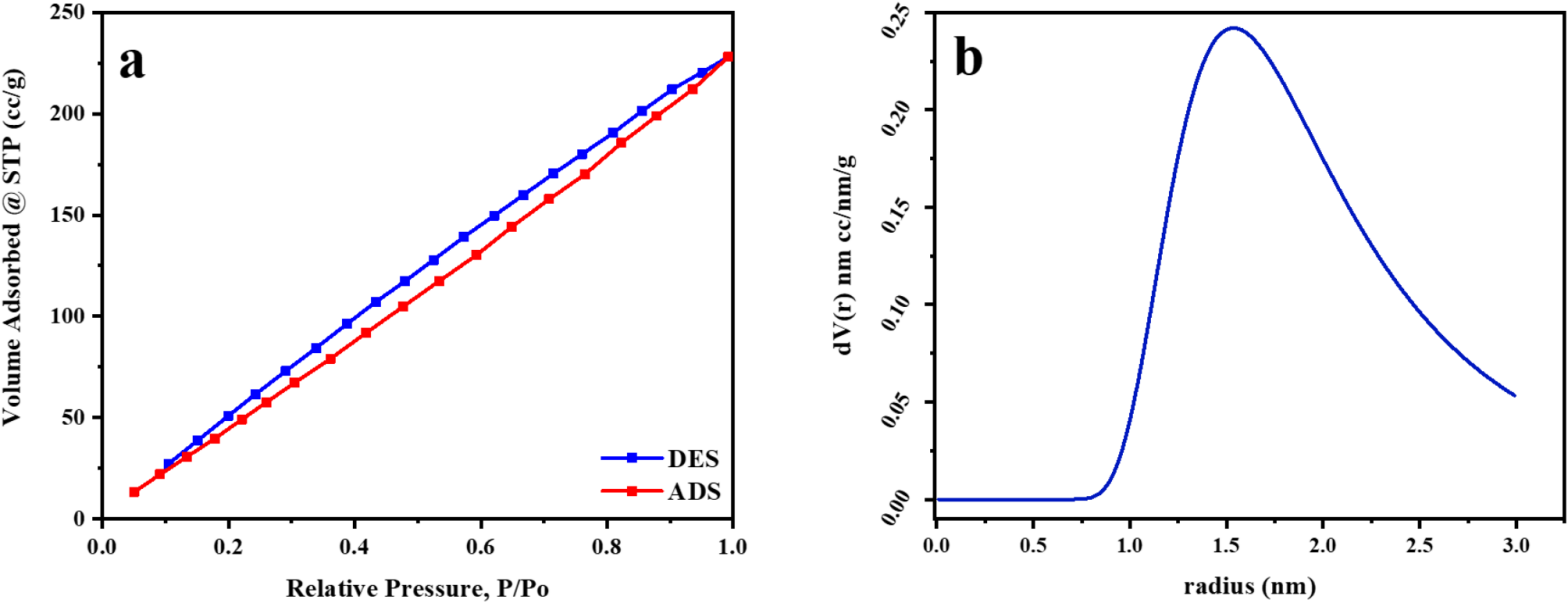
(**a**) Nitrogen adsorption-desorption isotherms of B, (**b**) The pore-size distribution for B.

**Fig. 5:**
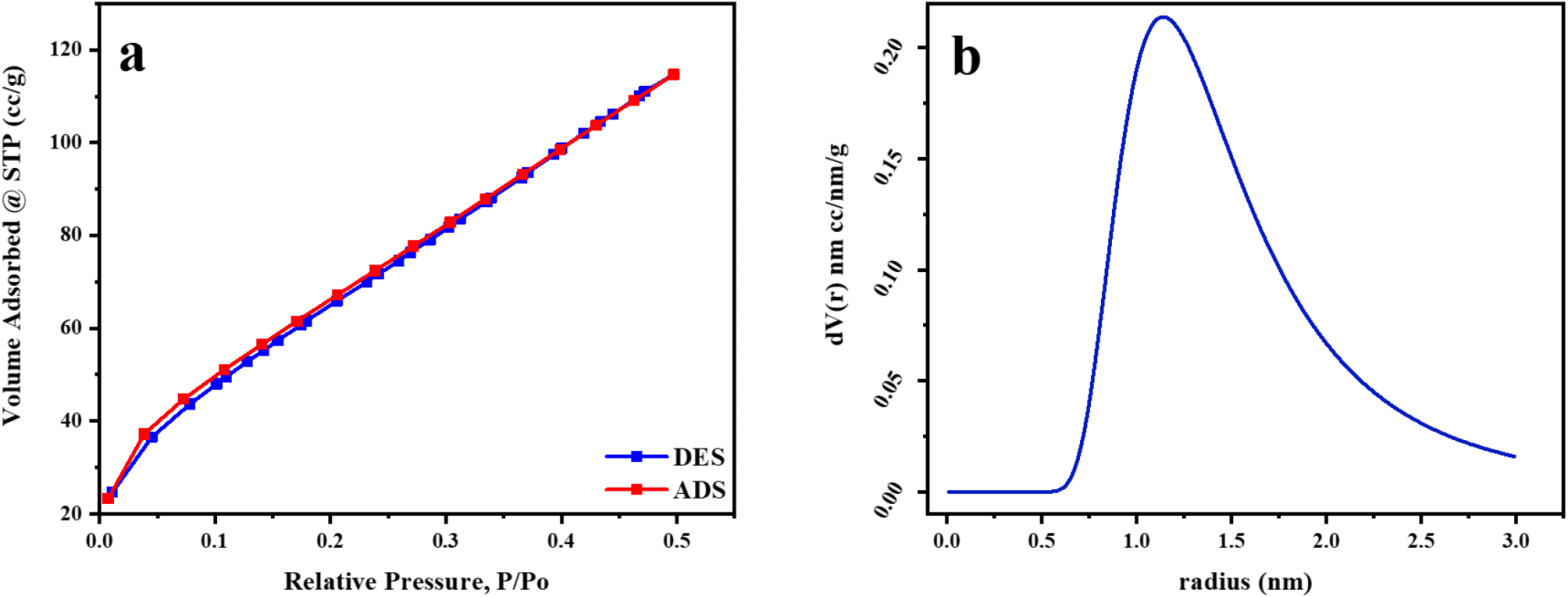
(**a**) Nitrogen adsorption-desorption of B(M), (**b**) The pore-size distribution for B(M).

The BET of natural bentonite is estimated to have 277.624 m^2^/g and 4.9118 nm particle radius. The BET surface area and the average particle radius were altered to 327.085 m²/g and 4.1691 nm, respectively. This demonstrates that the raw bentonite surface area is comparable to that of natural montmorillonite clay (475 m^2^ g^−1^)^53^. After treatment with microwave irradiation, the BET surface area expanded, hence enhancing the adsorption ability of Mn (VII) ions on the altered bentonite surface. The surface area of the MTB was approximately 18.0%more than that of the untreated bentonite B. The rise in average pore size from 1.28098 nm to 2.1616 nm supports the notion that the pore counts should be increased. It might also be attributed to the partial rearrangement of the skeleton of silica in the microwave-altered material, which indicated the formation of novel surface nucleation centers. Figures 4 and 5 depict the nitrogen adsorption and desorption isotherms and pore-size distribution for B and B(M).

#### SEM microscopy

**Fig. 6:**
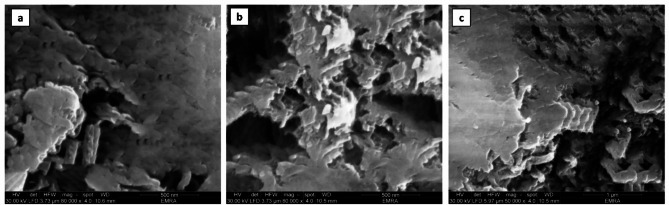
SEM image: (**a**) Natural bentonite, (**b**) microwave-treated bentonite (MTB), and (**c**) microwave-treated bentonite-Mn(VII) adsorption.

The SEM images are depicted in Fig. 6 which investigated the three cases of natural B, microwave-treated bentonite (MTB) and microwave-treated bentonite-Mn (VII) adsorption B(M)-Mn. Figure 6c explored the effect of Mn(VII) ions attachment from the solution onto the adsorbent. The adsorbent morphology exhibited a porous surface with particles of varying sizes. Particle aggregation of different sizes was shown by the MTB’s morphology, which showed a porous structure. The MTB’s pores are crucial because they significantly affect the uptake of metal ions from the solution into the MTB, which is seen as a black spot^54^. The interior structure of microwave-treated bentonite is significantly more layered and porous. The number of micro and mesopores rises, and the margins’ fringes disappear. The SEM images of MTB samples after the adsorption of Mn(VII) ions from solution are shown in Fig. 6c. The dark-field microscopy technique allows for the observation of placers of bright crystals (Mn(VII) on the dark background (alumosilicates), and many new crystals formed in the pores, with well-facetted crystals clearly visible on the smooth grain background.

### Point of zero charge of the MTB

**Fig. 7:**
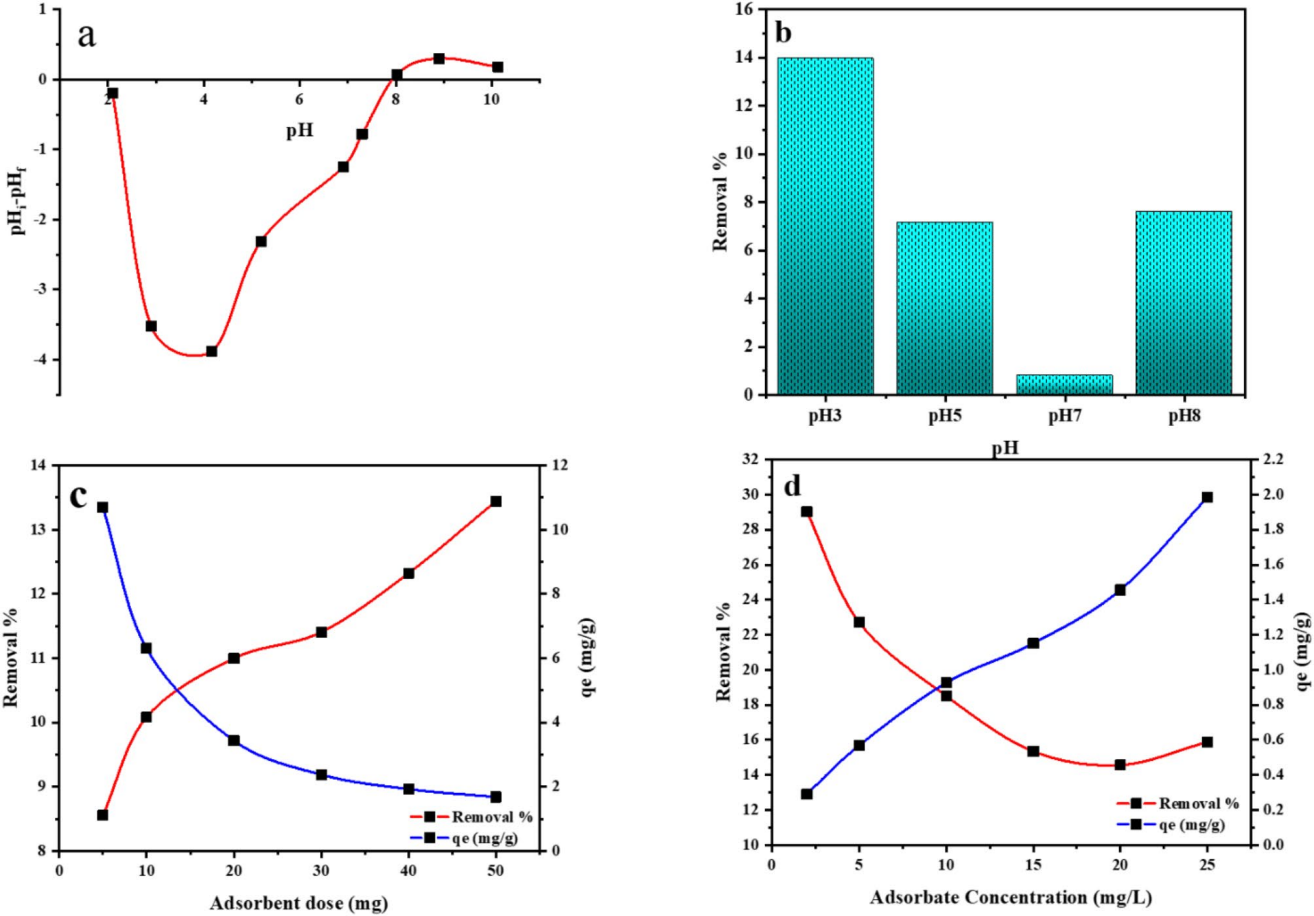
(**a**) the pH_pzc_ of the MTB and the effect of (**b**) pH MTB on the removal % of the Mn(VII) on the MTB, (**c**) sorbent dose, and (**d**) the sorbate concentration on the removal % of the Mn(VII) on the MTB as well as the corresponding adsorption capacity.

To observe the impact of varying pH values on the surface of the adsorbent, the pH level was altered from 2 to 10. Since the pH values influence the surface charge of the substance, this plays a vital function in the absorption process, which contributes to the accelerated elimination of metals. The point of zero charge (pH_PZC_) is the pH at which the charge on the surface of a substance is zero. If the pH is less than the value of pH_PZC_, then the substance has a positively charged surface and may adsorb anions, but if pH is larger than pH_PZC_, then the surface is negatively charged and can adsorb cations. By showing a link between pH_i_-pH_f_ and pH_i_, the PZC value can be estimated. It is derived by intersecting the resulting curve with the X-axis where the pH value is zero. Its pH value of 7.95 indicates that the surface of bentonite is positively charged at pH lower than 7.95 and negatively charged at pH higher than 7.95 (Fig. 7). And hence, there is a relationship between the pH_pzc_ and the adsorption capacity of an adsorbent; cation adsorption is favored where the sorbent surface is negatively charged (pH greater than pH_PZC_ and vice versa^55^.

### Factors that affect adsorption

#### The effect of pH

The Mn(VII) adsorption by MTB is pH-dependent. Underneath a pH range of 3 to 8, Mn (VII) removal percentage (R%) fluctuates significantly (Fig. 7b). Nevertheless, the greatest removal efficiency (14%) was only attained at pH 3. Because the pH of a solution may influence an adsorbent’s surface charge as well as the ionization state of the adsorbate in solution, and the extensiveness of separation of the functional groups on the adsorbent, a pH at 3 was selected in this investigation to the impact of the other operational conditions on adsorption.

At pH 3, the protonation degree is high, the negative charge of MnO_4_ ^−1^ was neutralized/ sorbed entirely on the exterior part of MTB, resulting in the highest adsorption capacity. This conclusion is supported by the pH_PZC_ finding of 7.95.

#### The effect of the dosage on the adsorbent

Various masses of the MTB ranging between (5–50 mg) were selected to investigate the relationship between the sorption and the sorbent mass. The elimination of Mn(VII) increases from 8.55 to 13.44% when the mass of MTB increases from 5 to 50 mg. Therefore, 50 mg was the optimal dose and was utilized in all adsorption experiments to confirm equilibrium achievement. Figure 7c further demonstrates that the Mn(VII) absorption decreases with increasing MTB dose at a constant Mn(VII) concentration and volume. This is due to the saturation impact of active sites resulting from particle interactions such as aggregation^55^. In contrast, the rise in Mn(VII) removal as MTB dosage was raised was owing to the increase in accessible MTB surface area in solution.

#### The effect of initial concentration and adsorption isotherms

The influence of starting metal concentration on the removal % of manganese ions was demonstrated in Fig. 7d. Results indicate that as the concentrations of Mn (VII) rise from 2 to 25 ppm, the removal efficiency decreases while the adsorption capacity increases. The Mn (VII) is removed by sorption on particular sites at lower concentrations, and the proportion between the starting concentration of Mn (VII) and the adsorption sites is high, Nevertheless, when the initial concentration of Mn(VII) is increased, this ratio drops and the particular sites become saturated. The elimination percentage then relies on the original concentration^56^. In this investigation, 25 mg/l of Mn(VII) was allotted for all the adsorption experiments.

The adsorption isotherms define the distribution of the adsorbate between the adsorbent as well as the solution based on the homogeneity of adsorbents, the kind of coverage, and the likelihood of contact between the adsorbate and adsorbent^58^. Several isotherm models, including the Freundlich, Temkin and Langmuir models, can describe the distribution of ions between the liquid phase and solid phase. Consequently, the sorption isotherm approaches are useful tools for comprehending the mechanism of interaction between MTB and Mn (VII). Using the equations of the models and the theoretical quantities of Mn(VII) sorption (mg/g) were calculated in order to study the most suited model.

#### Langmuir isotherm

Irving Langmuir proposed the Langmuir adsorption model in 1932 ^42^, with the following core assumptions: (a) the binding sites localized on the adsorbent surface are the key factor in adsorption process; (b) all adsorption sites on the adsorbent surface are similar; (c) the adsorbed particles form monolayer on the adsorbent surface; and the following equation illustrates the Langmuir adsorption model:

3 $$Ce / qe = 1/Kqmax + Ce /qmax$$

C_e_ is the adsorbate concentration at equilibrium (mg/ L).

q_e_ is the number of adsorbed molecules on the adsorbent surface at any time (mg/ g).

q_max_ shows the maximum adsorption capacity (mg/ g) and.

K_L_ represents the Langmuir constant (L/ mg).

**Fig. 8:**
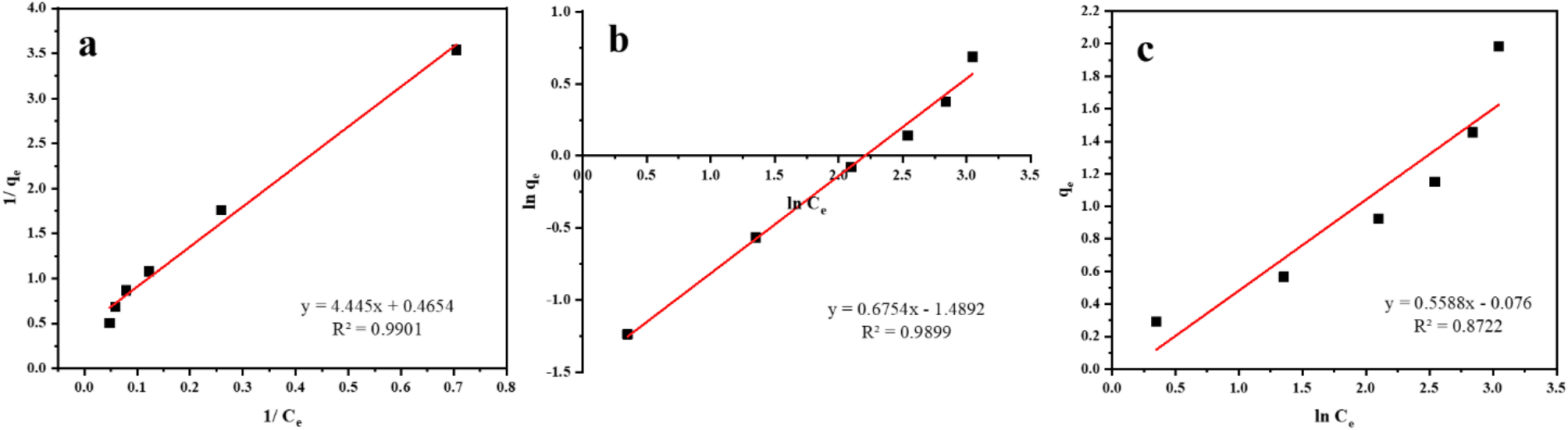
Adsorption isotherms of Mn(VII) onto MTB by (**a**) Langmuir, (**b**) Freundlich, and (**c**) Temkin.

The sorption results were analyzed using Langmuir isotherm (the linear form of the Eq. (3)(4), and the findings are depicted in Fig. 8a; Table 2. This indicates that the MTB has a maximum adsorption capability of 3.175 mg/g. The Langmuir isotherm requires the calculation of a dimensionless constant, separation factor (R_L_), given initial concentrations. The isotherm is theoretically unfavorable (if R_L_ > 1), linear (if R_L_ = 1), favorable (0 < R_L_ < 1), or reversible (if R_L_ = 0)^59,60^. The RL is determined by:

**Table 2:** Isotherms constants of Mn (VII) adsorption onto MTB.

Langmuir adsorption system		Freundlich adsorption system		Temkin adsorption system	
q_m_ (mg/g)	3.17	K_F_ (mg g^−1^)	4.4335	B (mg g^−1^)	0.5588
K_L_ (l/mg)	0.0562	*N*	1.4806	A (L/mg)	1.1457
R_L_	0.4158			b_T_ (J mol^−1^)	4436
R^2^	0.9901	R^2^	0.9899	R^2^	0.8722

4 $$RL =1 /(1+ K_LC_i)$$

where, C_i_ is initial Mn(VII) concentration and K_L_ (l/mg) is the Langmuir constant. The results of the Langmuir equilibrium parameter R_L_ (0.4158, i.e., 0 < R_L_< 1) demonstrate that the adsorption is favorable while the R^2^ of this model is 0.9901.

#### Freundlich isotherm

This model describes multilayer/ heterogeneous adsorption of molecules to the surface of adsorbents^43^. The following equation demonstrates this model:

5 $$log\, q_e = log K_F + 1/n log C_e$$

where:

q_e_ is the number of adsorbed molecules to the surface of sorbent at any time (mg/ g).

C_e_ is the concentration of Mn(VII) at equilibrium (mg/L).

K_F_ is the Freundlich exponent that describes the capacity of adsorption of the adsorbent towards the adsorbate (mg/ g).

n is the Freundlich constant (represents the sorption intensity), it indicates how the surface is heterogeneous and defines the distribution of the sorbed molecules on the adsorbent surface. A value of n greater than 1 implies preferential adsorption of molecules onto the surface of the adsorbent. A greater value of n indicates a greater intensity of adsorption. The sorption data were analyzed using the linear form of the Freundlich isotherm Eq. (5), and the findings are depicted in Fig. 8b; Table 2. The results indicate that *n* = 1.4806. This reveals that the adsorption is favorable and that there is a high concentration of Mn(VII) to be adsorbed on MTB. This model has an R^2^ of 0.9899.

#### Temkin model

Temkin and Pyzhev (1940)^44^ formulated an additional adsorption isotherm that considers the impact of adsorption heat that linearly decreases with the degree of occupation of the sorption sites. Because of the contact between the adsorbed molecules on the surface, adsorption heat decreases. The Temkin adsorption isotherm is as follows:

6 $$q_e = B ln A ln C_e Where B= RT/b_T$$

where:

q_e_ is the number of molecules adsorbed to the sorbent surface at any time (mg g^−1^).

C_e_ is the equilibrium concentration of Mn(VII) (mg L^−1^).

A is the adsorption constant at equilibrium corresponding to the maximum adsorption energy (L/mg).

B (mg/g): Temkin constant correlated to the adsorption heat.

R: The universal constant of gases (8.314 J/mol K).

T: The absolute temperature at 298 K.

b_T_: Constant of Temkin isotherm (J mol^−1^) related to the sorption heat.

The sorption results were analyzed by the linear form of the Temkin isotherm Eq. (4) and the results are shown in Fig. 8c; Table 2. The R^2^ value of this model is 0.8722.

Based on the linear regressions shown in Table 2, the R^2^ values of the three models suggest that the Langmuir isotherm best represents the adsorption of Mn(VII) on the MTB, as the experimental results best fit this model. This assumes that the MTB is heterogeneous and forms a multilayer covering of Mn(VII) species. The Freundlich and Temkin adsorption isotherms have smaller R^2^ values than the experimental data, hence they do not fit.

### Impact of shaking speed

**Fig. 9:**
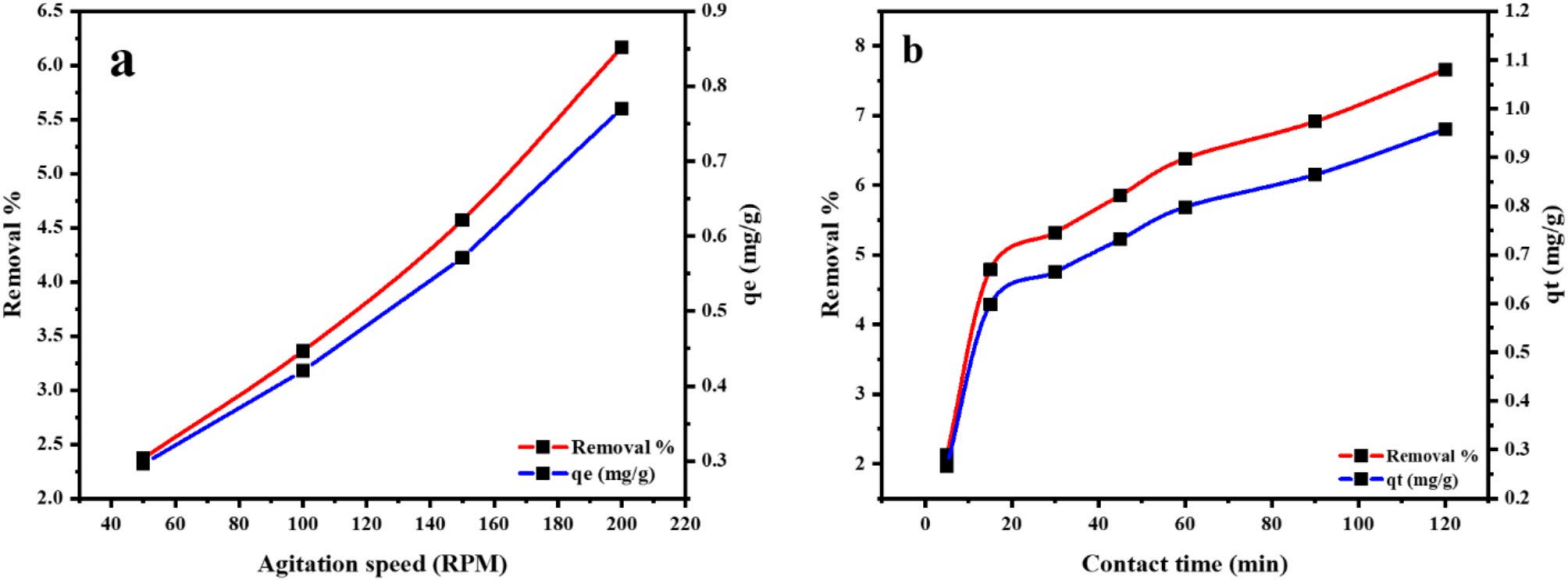
The impact of (**a**) the agitation speed and (**b**) the contact time on the removal % of the Mn(VII) on the MTB as well as the corresponding adsorption capacity.

Mn(VII) sorption of at varying agitation speeds (50 and 200 RPM) was shown in Fig. 9a. The removal percentage and adsorption capacity rise with increasing speed. Hence, a 200 RPM was the optimum agitation speed and was used with all adsorption experiments to confirm equilibrium achievement.

### Impact of contact time and kinetic models

Figure 9b depicts the effect of contact time on the Mn(VII) sorption on the MTB. About 7% of Mn(VII) ion was removed following a quick removal rate in the first 15 min, followed by a progressive increase until 120 min, when ~ 7% was removed.

## Kinetics of sorption

This dictates the pace of reaction, which defines the residence time and is one of the key parameters characterizing an adsorbent’s efficiency. Many independent mechanisms, such as film diffusion, bulk diffusion, intraparticle diffusion and chemisorption, can regulate the kinetics of ion sorption, either in parallel or in series^60^. To evaluate the Mn sorption on the MTB as well as the probable rate controlling steps (i.e., the optimal kinetic model for manganese elimination), the experimental results were modeled using Lagergren pseudo 1st order, pseudo 2nd order, and intraparticle diffusion models (Table 3).

**Table 3:** Kinetic parameters for the pentavalent Mn sorption on MTB.

Kinetic Models	Parameter	
q_e (exp)_		1.98
Pseudo-first order	q_e (cal)_	1.538
	K_1_	0.0037
	R^2^	0.8463
Pseudo- second order	q_e (cal)_ K_2_ h R^2^	1.047 0.0599 0.0657 0.9925
Intraparticle Diffusion	K_d_	0.1337
	C	0.0713
	R^2^	0.7515

### Lagergren kinetic model

The Lagergren kinetic equations are useful for determining an adsorption performance of the adsorbent. Lagergren pseudo 1st order and pseudo 2nd order kinetics are typically linearly written as Eqs. (7) and (8), respectively.

7 $$ln (q_e - q_t ) = ln q_e - K_1t$$

8 $$t / q_t = 1 / K_2 q_e^2+ t/q_e$$

Where:

 and (mg/g) are the adsorption capacity of metal ions at time (min) and at equilibrium state, respectively.

K_1_ (min^−1^) and K_2_ (g mg^−1^ min^−1^) are the Lagergren rate constants of adsorption of the pseudo 1st order and the pseudo 2nd order kinetic models.

#### a) Pseudo 1st order kinetic model

**Fig. 10:**
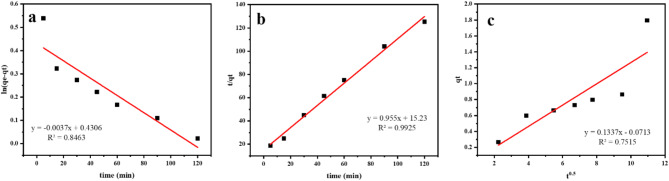
Adsorption kinetic models of Mn(VII) onto MTB by (**a**) pseudo-first order, (**b**) pseudo-second order, and (**c**) intraparticle diffusion.

According to the Lagergren pseudo 1st order model, the site occupation rate is related to the number of empty sites. The linearized pseudo 1st order plot of the Mn(VII) sorption in starting concentration (25 ppm) onto the MTB is presented in Fig. 10a. Using the slope and intercept of the plots of ln (q_e_- q_t_) over time, the pseudo 1st order rate constant K_1_ and equilibrium adsorption capacity q_e_ for the Mn(VII) ions were calculated (Table 3). The coefficient of regression R_2_ for the adsorption system is 0.8463.

#### b) Pseudo 2nd order kinetic model

The pseudo 2nd order model is predicated on the notion that adsorption is a 2nd process. Therefore, the site occupation rate is proportional to the number of empty sites squared^62^. The applicability of the pseudo 2nd order model was evaluated using a linear plot of t/q_t_ vs. t (Fig. 10b) with a slope of 1/q_e_; the value of K_2_ was determined from the intercept of the plot (Table 3). From the equation, the initial sorption rate (h) can be derived.

9 $$h = K_2 q_e^2$$

The regression coefficient R^2^ of the adsorption system is 0.9925.

#### c) intraparticle diffusion

The intraparticle diffusion model determined the mechanism of diffusion^63^ where the ions are carried from the aqueous solution to the sorbent surface. If the particles are porous, they can diffuse inside the particles. According to^62^, the intraparticle diffusion equation is:

10 $$q_t =C + K_d t^{0.5}$$

Where: where q_t_ (mg/g) is the adsorbed amount at time (h), C is the intercept and K_d_ (mg/g.min^1/2^) is the intraparticle diffusion rate constant that is related to the thickness of boundary layer. A linear relationship that passes through the origin in the q_t_ versus t^1/2^ plot indicates that the single rate determining step is intraparticle diffusion (C = 0). Values of C and q_t_ were obtained from the intercept and the slope of the plot (Table 3). The intra-particle diffusion plot is shown in (Fig. 10c). Intraparticle Diffusion plot shows R^2^=0.7515.

Comparing the correlation coefficients (Table 3) revealed that the pseudo-second order model provided excellent fit to the experimental results for the Mn(VII) chemisorption mechanism. Moreover, the computed q_e_ values (q_ecal_) derived from the pseudo 2nd order model are marginally lower than the experimental q_e_ value (q_eexp_). Despite the reasonable match of the pseudo 2nd order model, it was observed that the differences between the experimental as well as appraised q_e_ may be attributable to a temporal lag, external resistance control or boundary layer at the beginning of the sorption process^64^. In contrast to the pseudo-second order model, the qecal of the pseudo-first order model for Mn(VII) ion adsorption was more in line with the actual value. Additionally, the intra-particle diffusion model’s R2 values showed a moderate correlation for Mn(VII) ions (0.7515), indicating that the adsorption of Mn(VII) on the MTB could not be explained by the intra-particle diffusion mechanism.

### Thermodynamics of Mn (VII) adsorption

**Fig. 11:**
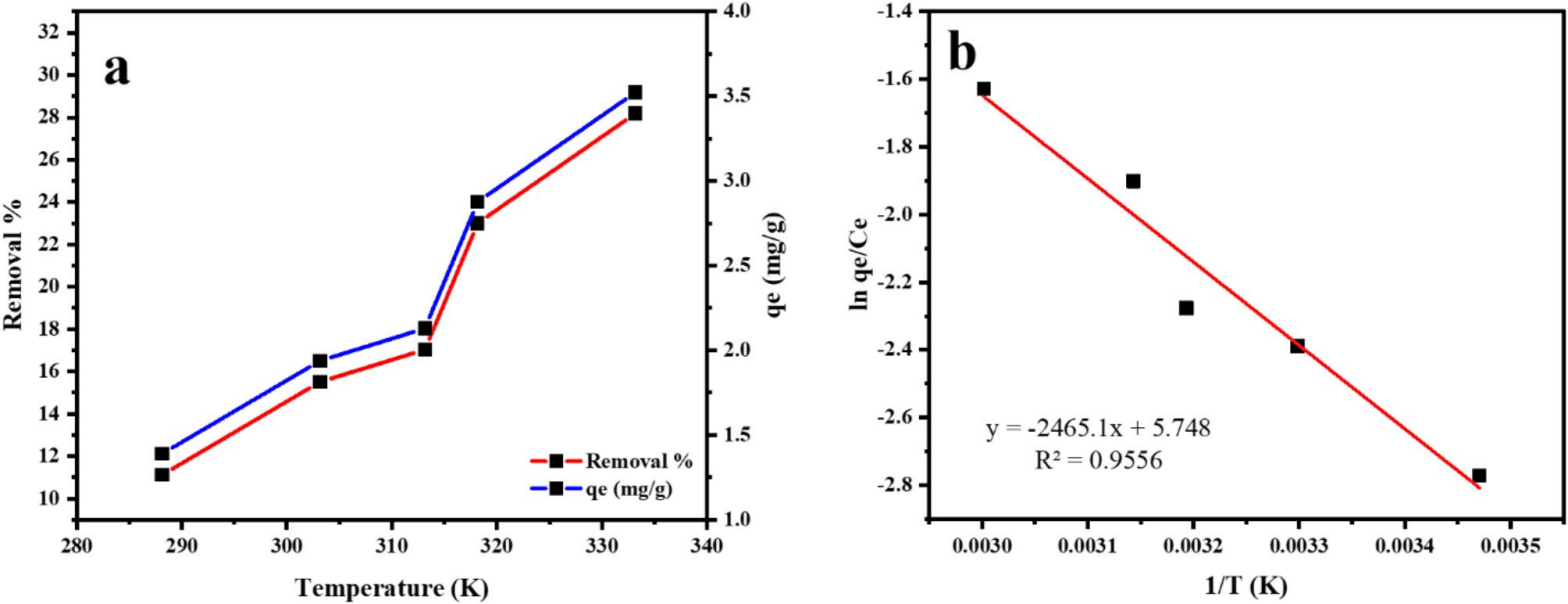
**A**) The effect of solution temperature on the removal % and the adsorption capacity of Mn(VII) on MTB, and **b**) Thermodynamic plot for the Mn(VII) sorption on MTB.

The effect that the temperature of the solution has on both the proportion of Mn(VII) ions that are removed from the solution and the amount of adsorption capacity that the MTB is shown in Fig. 11a. It has been observed that as the temperature rises, the percentage of Mn(VII) ions that are removed also rises. In order for the Mn(VII) to pass the energy barrier between the ions and MTB, they must gain more energy as the temperature rises. This results in the formation of more extra sites on the adsorbent surface. The Gibbs free energy (G° in kJ/mol), alterations in enthalpy (H° in J mol-1), and changes in entropy (S° in J K-1 mol-1) were calculated using the Van’t Hoff equation in Eqs. 11 and 12^65^:

11 $$\normalsize \triangle{G^o}=\triangle{H^o}-{T}\triangle{S^o}$$

12 $$ln K_c = - (\triangle{H^o /RT})+(\triangle{S^o/R})$$

13 $$K_c = q_e/C_e$$

where:

(T = temperature in K, R = the ideal gas constant (8.314 J/molK), K_c_ = the distribution coefficient, qe = the concentration of metal ion adsorbed in mg/g, and C_e=_ the concentration of the metal ion in solution after equilibrium in (mg/L). ΔH° and ΔS° were obtained from the slope and intercept of the lnK_c_ and 1/T plot (Fig. 11b). The findings of the investigation of the thermodynamic characteristics of manganese adsorption on MTB at temperatures of 288, 303, 313, 318, and 333 K are shown in Table 4. At each of the temperatures tested, the ΔG° for Mn sorption by MTB was negative, ranging from (−34265 to −36415 kJ/mol). Based on these findings, it appears that manganese adsorption occurred spontaneously. The enthalpy values for the adsorption of MTB were − 20495 at degrees Celsius (kJ mol-1). The fact that the value of enthalpy was negative suggests that manganese adsorption on MTB represents an exothermic reaction. Hypothetically, ΔH° values implied that hydrogen bonding (2 to 40 kJ mol^−1^), Van der Waals forces (4 to 10 kJ mol^−1^), chemical bonding forces (60 kJ mol^−1^), and ligand exchange (40 kJ mol^−1^) were the main adsorbent- adsorbate attraction^66^. In this study, Mn sorption by MTB was governed by the forces of chemical bonding. ΔS° values (positive) indicated randomness on the active sites of the MTB.

**Table 4:** Thermodynamic parameters for the Mn (VII) sorption on MTB.

T (K)	K_c_	ΔG° (kJ/mol)	ΔH° (kJ/mol)	ΔS° (J/mol.K)	*R* ^2^
288	0.06	−34,265	−20,494	48	0.9556
303	0.09	−34,982			
313	0.10	−35,459			
318	0.15	−35,698			
333	0.20	−36,415			

#### Evaluation of MTB reusability

**Fig. 12:**
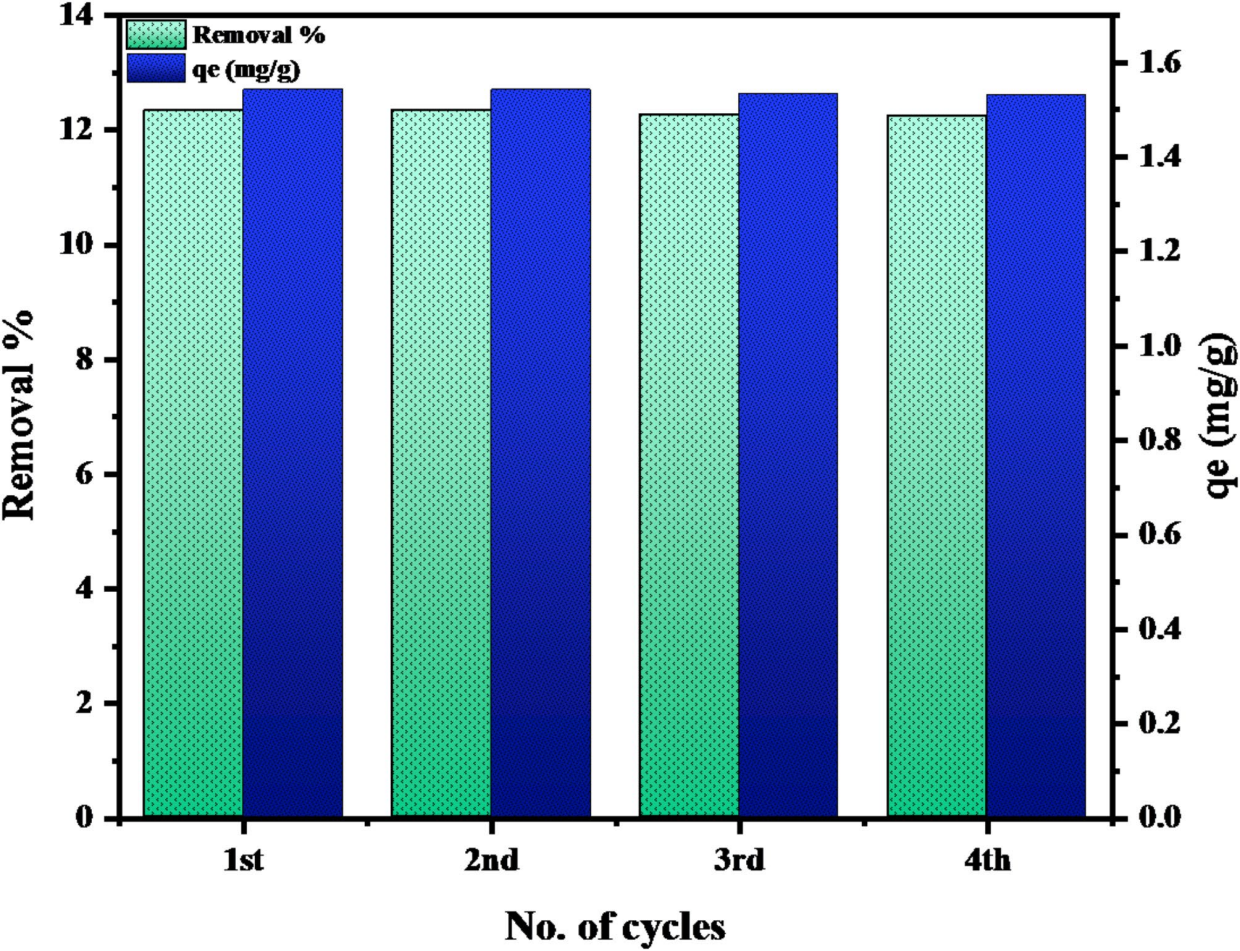
Reusability of MTB.

The regeneration and reprocessing of adsorbents is a crucial economic factor, as it impacts production costs^67^. The instantaneous attraction between the pentavalent manganese ions and the MTB surface was regained by regenerating the Mn(VII)-adsorbed MTB. The reusability of MTB in subsequent cycles was examined by desorbing Mn(VII) from loaded MTB with 0.15 M HCl and then reusing the regenerated MTB to adsorb Mn (VII). Desorption of Mn(VII) was performed in a shaker in which loaded MTB was combined with diluted HCl, H_2_SO_4_ or citric acid and agitated in a baker before being washed with distilled water. After desorption, regenerated MTB was used for adsorption once more. As shown in Fig. 12, the uptake affinity of the MTB decreased from 12.34 to 12.25 after four consecutive cycles. The obtained patterns of manganese uptake and desorption in numerous cycles validated the reusability of MTB for Mn(VII) removal.

#### Theoretical adsorption study

**Fig. 13:**
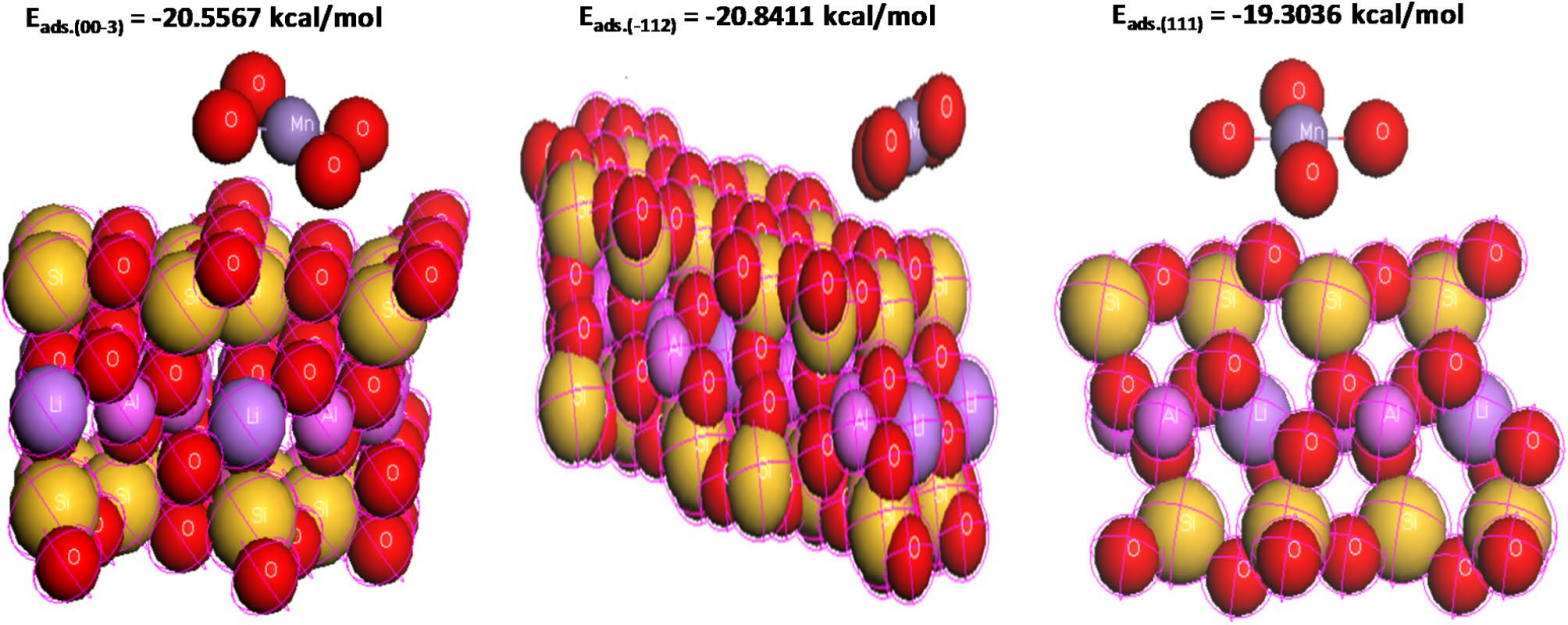
Modes of adsorption permanganate on bentonite planes.

In Monte Carlo simulation, MDs were performed on a system comprising permanganate, and bentonite (00–3, −112 and 111) surfaces. We selected three observed planes in the XRD spectrum to investigate the adsorption process. The simulation process of the Monte Carlo model tried to obtain the lowest values of energy. A highly helpful technique for improving comprehension of the adsorption mechanisms with reference to structural and energetic information is molecular dynamics (MD). Both the conformational rearrangements of molecules and the interactions between the various parts of a molecular system can be used to determine this truth. In this study, the molecular dynamics approach is used to theoretically analyze the adsorption process of Mn(VII) onto MTB in order to evaluate the intermolecular interactions and affinity of the studied Mn(VII) toward MTB^68^.The overall energy of the surface-permanganate configuration is represented by the descriptors in Table 5, which are the sum of the rigid adsorption energy, adsorbate energies, and deformation energy; the substrate energy is assumed to be zero. The adsorption energy was the total of the rigid adsorption energy and the deformation energy. The energy needed (or released) for the unrelaxed permanganate ions to be adsorbed on the substrate is known as the stiff adsorption energy. The energy of substrate–adsorbate configurations in which the permanganate has been eliminated is defined by the deformation energy, which reports the energy released when the adsorbed permanganate is loosened on the substrate surface dEad/dNi. Permanganate exhibits the highest total energy and stiff adsorption energy, as shown in Table 5, which validates the experimental findings. Figure 13 displays the optimal adsorption configurations and the intimate contact between the permanganate and bentonite surfaces. Out all the estimated planes, the (−112) plane surface has the best adsorption energy. The spontaneity of the adsorption process is demonstrated by the negative energy value, as we have observed.

**Table 5:** The outputs and descriptors calculated by Monte Carlo simulation for adsorption of permanganate on bentonite (00–3, −112 and 111) planes.

planes	Total energy kJ mol^−1^	Adsorption energy kJ mol^−1^	Rigid ad energy kJ mol^−1^	Deformation energy kJ mol^−1^	Atomistic : dEad/dNi
(002)	−5.44	−0.164	−5.444	−15.11	−20.55
(−112)	−5.72	−20.84	−5.73	−15.11	−20.84
(111)	−4.19	−19.30	−4.19	−15.11	−19.30

### Working mechanism

As illustrated in Figs. (8–11), the adsorption mechanism can be investigated using a variety of methods based on the kinetics, isotherms, and thermodynamic investigations. The adsorption mechanism seems to have involved electrostatic interactions between negatively charged MnO_4_^−^species and positively charged adsorbent surfaces. Furthermore, the formation of an O.M bond or the sharing of free electron pairs between the metal atom and the surface oxygen atom led to covalent bonding. The hydrogen bond between the surface oxygen atoms of hydrated metal ions and the ion-exchange interaction may also be important components of the adsorption mechanism^69,70^. The theory of surface chemistry states that MTB particles and metal ions are absorbed by the metal ions’ interaction with the surface groups. The MnO^−^ _4_ species and protonated species of the MTB adsorbent had the highest uptake percentages because of the many attraction forces between MTB and permanganate (MnO_4_^−^) species, including electrostatic attraction forces, caused by the highly protonated exterior surface of MTB (pH 3.0). The highest percentage of heptavalent manganese removal was at pH 3. In certain situations, the presence of the C–O group may cause Mn^+7^ to be reduced to Mn^+2^ in an acidic solution^69^.

## Conclusion and recommendations

The Mn(VII) sorption on microwave-treated bentonite (MTB) has been examined in this study. The findings indicate that MTB is a low-priced adsorbent for removing pentavalent manganese from aqueous solutions. Microwave-treated bentonite had a surface area that was approximately 18.0% larger than raw bentonite. The rise in average pore size from 1.28098 nm to 2.1616 nm supports the notion that the number of pores should be increased. The adsorption of pentavalent manganese is dependent on the MTB dose, the solution’s pH, the initial concentration, the temperature, and the agitation time. The pseudo 2nd order equation describes the heptavalent manganese sorption on MTB more accurately than the pseudo 1st order equation and the intraparticle diffusion model. This facilitates the chemisorption process. In addition, based on enthalpy change measurements, the Mn(VII) sorption on MTB is an exothermic and spontaneous reaction. MTB exhibited the same removal percentage and absorption on Mn(VII) ions in two consecutive cycles, demonstrating its suitability for repeated Mn(VII) removal. The utilized MTB might be used in batch and column investigations including various single ions, as well as binary, ternary, and multiple ions’ systems. The spontaneity of the adsorption process was observed during the stimulation study.

## Data Availability

Data is provided within the manuscript.

## Abbreviations

APHA 2022: American Public Health Association 2022
BET: Brunauer − Emmett − Teller
BJH: Barrett − Joyner − Halenda
FTIR: Fourier Transform Infra-Red
LOI: Loss Of Ignition
Mn(VII): manganese hepta hydrate
MTB: microwave-treated bentonite
MWCNT: multi wall carbon nanotubes
pHPZC: point of zero charge
SEM: Scanning Electron Microscopy
TEM: Transmittance Electron Microscopy
XRF: X-ray Fluorescence
